# Supplementary material for: Reducing the Bitter Taste of Pharmaceuticals Using Cell-Based Identification of Bitter-Masking Compounds
Source: Pharmaceuticals (Basel). 2022 Mar 7;15(3):317. doi: 10.3390/ph15030317 (PMC8953435; doi:10.3390/ph15030317)
Supplement: Supplementary file 1 [file pharmaceuticals-15-00317-s001.zip › pharmaceuticals-1583865-supplementary.pdf]

Communication

Reducing the Bitter Taste of Pharmaceuticals Using Cell-Based Identification of Bitter-Masking Compounds

Leopoldo Raul Beltrán [1,†], Sonja Sterneder [1,†], Ayse Hasural [1], Susanne Paetz [2], Joachim Hans [2], Jakob Peter Ley [2] and Veronika Somoza [1,3,4,*]

[1] Department of Physiological Chemistry, University of Vienna, 1090 Vienna, Austria; leopoldo_beltran@outlook.de (L.R.B.); sonja.sterneder@univie.ac.at (S.S.); a01347723@unet.univie.ac.at (A.H.)
[2] Symrise AG, Ingredient Research Flavor & Nutrition, 37603 Holzminden, Germany; susanne.paetz@symrise.com (S.P.); joachim.hans@symrise.com (J.H.); jakob.ley@symrise.com (J.P.L.)
[3] Leibniz-Institute of Food Systems Biology at the Technical University of Munich, 85354 Freising, Germany
[4] Nutritional Systems Biology, Technical University of Munich, 85354 Freising, Germany
* Correspondence: veronika.somoza@univie.ac.at; Tel.: +43-1-4277-70601
† These authors have contributed equally to this research and share first authorship.

Figure S1:

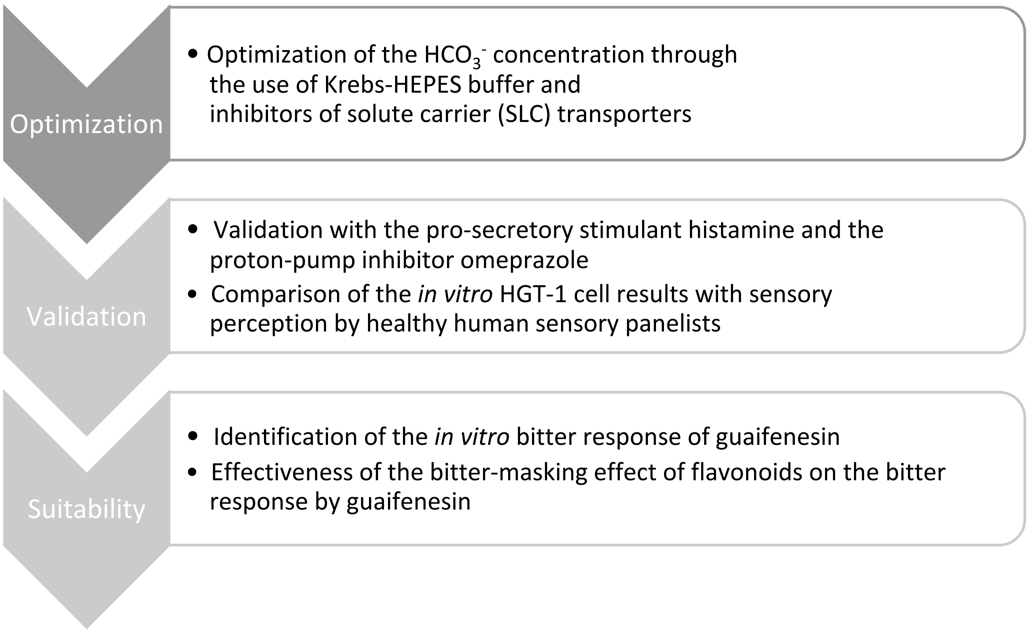

Figure S1: Experimental approach.

Figure S2:

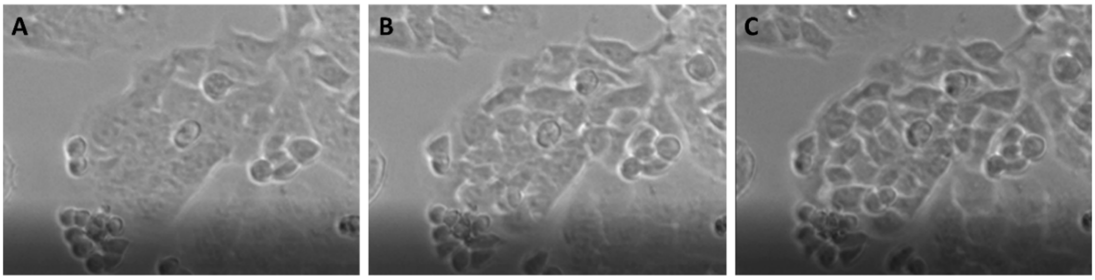

Figure S2. Histamine priming of HGT-1 cells. Showing the morphological changes of HGT-1 cells exposed to histamine (molecular structure as inset) 10 μM for 0 min (**A**), 30 min (**B**) and 60 min (**C**). Light microscopy, ×10 magnification.
